# Tetrabromobisphenol A Disturbs Brain Development in Both Thyroid Hormone-Dependent and -Independent Manners in *Xenopus laevis*

**DOI:** 10.3390/molecules27010249

**Published:** 2021-12-31

**Authors:** Mengqi Dong, Yuanyuan Li, Min Zhu, Jinbo Li, Zhanfen Qin

**Affiliations:** 1 State Key Laboratory of Environmental Chemistry and Ecotoxicology, Research Center for Eco-Environmental Sciences, Chinese Academy of Sciences, Beijing 100085, China; dongmengqi029@163.com (M.D.); yyli@rcees.ac.cn (Y.L.); minzhu21@163.com (M.Z.); chnlijinbo@163.com (J.L.); 2 University of Chinese Academy of Sciences, Beijing 100049, China

**Keywords:** tetrabromobisphenol A, *Xenopus laevis*, brain development, thyroid hormone, biphasic concentration-response

## Abstract

Although tetrabromobisphenol A (TBBPA) has been well proven to disturb TH signaling in both in vitro and in vivo assays, it is still unclear whether TBBPA can affect brain development due to TH signaling disruption. Here, we employed the T3-induced *Xenopus* metamorphosis assay (TIXMA) and the spontaneous metamorphosis assay to address this issue. In the TIXMA, 5–500 nmol/L TBBPA affected T3-induced TH-response gene expression and T3-induced brain development (brain morphological changes, cell proliferation, and neurodifferentiation) at premetamorphic stages in a complicated biphasic concentration-response manner. Notably, 500 nmol/L TBBPA treatment alone exerted a stimulatory effect on tadpole growth and brain development at these stages, in parallel with a lack of TH signaling activation, suggesting the involvement of other signaling pathways. As expected, at the metamorphic climax, we observed inhibitory effects of 50–500 nmol/L TBBPA on metamorphic development and brain development, which was in agreement with the antagonistic effects of higher concentrations on T3-induced brain development at premetamorphic stages. Taken together, all results demonstrate that TBBPA can disturb TH signaling and subsequently interfere with TH-dependent brain development in *Xenopus*; meanwhile, other signaling pathways besides TH signaling could be involved in this process. Our study improves the understanding of the effects of TBBPA on vertebrate brain development.

## 1. Introduction

Tetrabromobisphenol A (TBBPA) is usually used as brominated flame retardants in various products, including printed circuit boards, adhesives, coatings, etc. [1]. The extensive production and use of TBBPA have led to wide existence in the environment, especially in China. It was reported that the TBBPA concentration in surface water from Lake Chaohu (China) reached 4.87 μg/L [2]. Monitoring studies show that TBBPA is frequently detected in human samples, such as urine, blood, breast milk, and adipose tissue, including samples from pregnant women [3,4,5]. Although the European Union (EU) approved TBBPA as no significant adverse effects for human health based on a risk assessment of TBBPA following a series of standard guideline-compliant toxicity tests [6,7], there is growing evidence that TBBPA could exert certain adverse effects, including carcinogenicity, in laboratory animals [8]. Especially, TBBPA has been shown to interfere with thyroid hormone receptor (TR), thereby altering TR-mediated gene transcription in vitro assays, indicating its TH signaling disrupting activity [9,10]. Importantly, several research groups, including ours, reported that TBBPA antagonized TH-induced metamorphic development in amphibians and even inhibited spontaneous metamorphosis at the metamorphic climax, along with changes in the expressional levels of TH-response genes, demonstrating the TBBPA’s actions on TH signaling in vivo [11,12,13].

Given the key roles of TH in brain development, we suspect that TBBPA could disturb TH-dependent brain development in vertebrates. However, few studies have addressed this issue. In fact, whether or not TBBPA can exert adverse effects on the nervous system is still controversial. Based on a series of standard guideline-compliant toxicity tests, the EU concluded that TBBPA is not a neurotoxicant for both adult and developing mammals [6]. Kacew and Hayes [14] declared that TBBPA has little neurotoxicity to vertebrates, including humans, considering limited neurobehavioral effects and its limited presence in the brain. However, there is increasing evidence that TBBPA could exert adverse effects, such as increased anxiety and reduced social behaviors, in laboratory mammals and fish [15]. Nevertheless, no data has addressed whether or not the neurotoxic effects of TBBPA are associated with its TH signaling disruption. Indeed, it is challenging to employ rodents to reveal whether chemicals could exert neurotoxic effects through TH signaling due to the involvement of multiple signaling pathways. In contrast to rodents, amphibians undergo metamorphic development, including brain remodeling, mainly regulated by TR-mediated TH signaling that activates the expression of a series of TH-response genes. Notably, TH addition in water can induce precocious metamorphosis of pre-metamorphic tadpoles, characterized by upregulated expression of TH-response genes and subsequent cellular, histological and morphological changes of various organs [16,17]. Thus, the metamorphic development of amphibians, especially the model species *Xenopus laevis*, acts as an excellent model to dissect TH signaling and TH-dependent development [17,18]. Previously, we developed the T3-induced *Xenopus* metamorphosis assay (TIXMA) for evaluating TH signaling disruption and subsequent effects on TH-dependent development [19,20]. Specifically, TH-response gene expression is used as endpoints for TH signaling disruption, while morphological, histological, and cellular parameters are employed for evaluating the effects on TH-dependent development. Since the TIXMA involves the parameters of brain remodeling, it offers a great chance to evaluate the interference of chemicals on TH signaling and subsequent effects on brain development [21]. In our previous study investigating TH signaling disruption of TBBPA [13], we noticed that TBBPA appeared to have an effect on T3-induced brain morphological alternations in *Xenopus*.

The aim of this study was to determine whether low concentrations of TBBPA could interact with TH signaling in *Xenopus* brains and alter TH-dependent brain development. Following the TIXMA we established previously [13,22], pre-metamorphic tadpoles were treated with TBBPA in the absence or presence of T3. On day two after exposure, TH-response gene expression was measured for evaluating TH-signaling disruption in the brain, while on day four, brain morphological and cellular changes were examined for evaluating the effects on TH-dependent brain development. Additionally, the effects of TBBPA on spontaneous TH-dependent brain development were investigated to verify the results observed in the TIXMA. This study is expected to enhance our understanding of the neurodevelopmental effects of TBBPA in vertebrates.

## 2. Results

### 2.1. TBBPA Affects T3-Induced TH-Response Gene Expression in Xenopus laevis Brains

**Figure 1:**
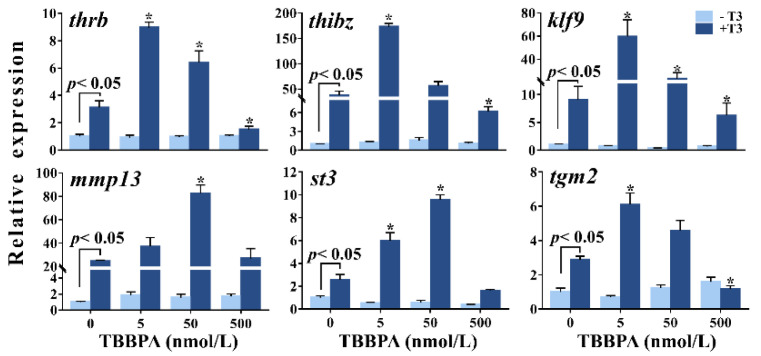
The relative expression of six thyroid hormone-response genes in brains of premetamorphic *Xenopus laevis* tadpoles following two-day exposure to tetrabromobisphenol A (TBBPA) in the absence or presence of 1 nmol/L T3. Data are shown as mean ± SEM. * indicates significant differences between TBBPA + T3 treatment and T3 treatment (*p* < 0.05).

As expected, two days of T3 treatment dramatically upregulated the expression of all TH-response genes (*klf9*, *thrb*, *thibz*, *mmp13*, *tgm2*, and *st3*) in *Xenopus* brains (*p* < 0.05) (Figure 1). TBBPA exposure alone had no effects on the expression levels of these genes. In the presence of T3; however, 5 nmol/L and/or 50 nmol/L TBBPA resulted in higher expression of these TH-response genes, whereas the highest concentration caused lower expression of *thrb*, *thibz*, *klf9*, and *tgm2*, compared with T3 treatment. Altogether, these observations show that 5–500 nmol/L TBBPA affected T3-induced TH-response gene expression in a biphasic concentration-response manner.

### 2.2. TBBPA Alters Brain Morphology in the Absence and Presence of T3

**Figure 2:**
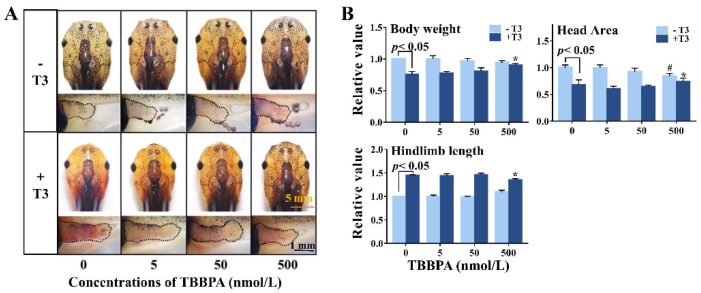
Morphological changes of premetamorphic *Xenopus laevis* following four-day exposure to tetrabromobisphenol A (TBBPA) in the absence or presence of 1 nmol/L T3. (**A**) Representative morphology of the head and the hindlimb of exposed tadpoles. (**B**) Quantitative analysis for body weight, hindlimb length, and head area of exposed tadpoles. Data are shown as mean ± SD. # indicates significant differences between TBBPA treatment and solvent control treatment (*p* < 0.05). * indicates significant differences between TBBPA + T3 treatment and T3 treatment (*p* < 0.05).

After four days of treatment, the 500 nmol/L TBBPA-treated tadpoles had longer hindlimbs with more obvious toes and smaller head area than controls, showing that 500 nmol/L TBBPA promoted pre-metamorphic development (Figure 2A). As expected, T3 treatment induced precocious metamorphosis, characterized by body weight loss, hindlimb growth, head shrinkage (Figure 2B), and brain remodeling. Following co-treatment with T3 and 50–500 nmol/L TBBPA, morphological changes exhibited a concentration-dependent weakening trend compared with T3 treatment, indicating that 50–500 nmol/L TBBPA antagonized T3-induced metamorphosis in a concentration-dependent manner. A slight effect was found at the lowest concentration of TBBPA on T3-induced metamorphosis in *Xenopus* brain.

**Figure 3:**
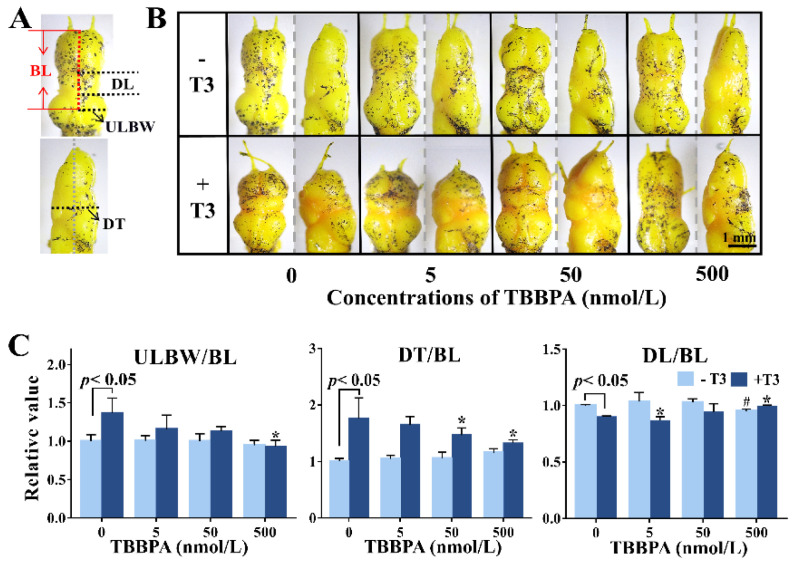
Brain morphological changes of premetamorphic *Xenopus laevis* following four-day exposure to tetrabromobisphenol A (TBBPA) in the absence or presence of 1 nmol/L T3. (**A**) Schematic description of measurement parameters. DL for diencephalon length, ULBW for unilateral brain width, BL for brain length, and DT for diencephalon thickness. (**B**) Representative morphology of the elevation view and side view of the brain. (**C**) Quantitative analysis result of brain parameters. Each parameter was normalized by the mean value of the solvent control tadpoles. Data are shown as mean ± SD. # indicates significant differences between TBBPA treatment and solvent control treatment (*p* < 0.05). * indicates significant differences between TBBPA + T3 treatment and T3 treatment (*p* < 0.05).

In terms of brain morphological changes, mainly longitudinal shortening as well as dorsoventral and lateral broadening, T3 treatment induced dramatic brain remodeling, particularly in the diencephalon. We characterized brain remodeling with ULBW/BL, DL/BL, and DT/BL (Figure 3A). Compared with controls, T3-treatment resulted in increases in ULBW/BL and DT/BL but decreased in DL/BL (Figure 3C). Interestingly, 500 nmol/L TBBPA treatment alone also decreased DL/BL. In 50–500 nmol/L TBPPA and T3 co-treatment groups, tadpoles exhibited lower ULBW/BL and DT/BL values and higher DL/BL values in comparison with T3-treated animals. As a whole, 50–500 nmol/L TBBPA appeared to concentration-dependently antagonize T3-induced increases in ULBW/BL and DT/BL but decrease in DL/BL. In contrast, 5 nmol/L TBBPA combined with T3 resulted in a significant decrease in DL/BL than T3 treatment, displaying an agonistic effect on T3 action. Collectively, 5–500 nmol/L affected T3-induced brain development in a biphasic concentration-response manner.

### 2.3. TBBPA Affects Cell Proliferation in the Telencephalon in the Presence of T3

**Figure 4:**
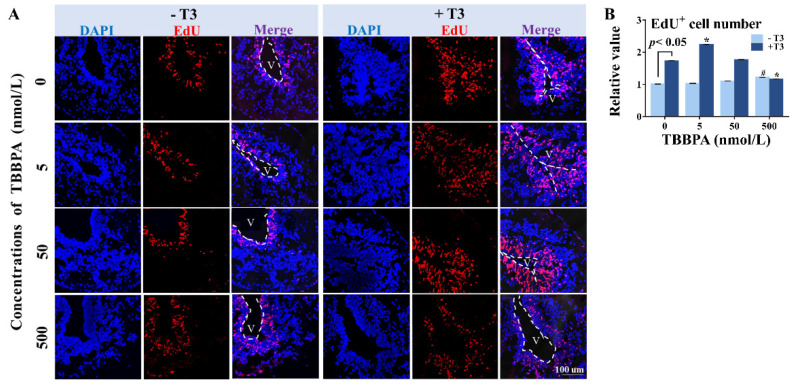
EdU-labeled cell proliferation in telencephalons of pre-metamorphic *Xenopus laevis* following 4-day exposure to tetrabromobisphenol A (TBBPA) in the absence or presence of 1 nmol/L T3. (**A**) Immunofluorescence images of telencephalons at the end of the T3-induced metamorphosis assay. EdU label (red) and DAPI (blue). (**B**) The ratio of EdU positive cells number. Three animals were analyzed for each treatment. V: ventricle. Parameter was normalized by the mean value of the solvent control. Data are shown as mean ± SD. # indicates significant differences between TBBPA treatment and solvent control treatment (*p* < 0.05). * indicates significant differences between TBBPA + T3 treatment and T3 treatment (*p* < 0.05).

EdU-labeled cells were observed in the subventricular zone (SVZ) in the telencephalon, showing active cell proliferation in this zone (Figure 4A). Some of the EdU-labeled cells were also present in the surrounding of the SVZ, meaning their migration from the SVZ because cell proliferation is known to only occur in the SVZ [23]. Corresponding to brain morphological changes, T3 treatment for four days caused more EdU-labeled cells in the SVZ compared with controls, and some EdU-labeled cells migrated from the SVZ, along with shrunk ventricles, indicating that T3 stimulated cell proliferation and subsequent migration from the zone. Increased cell proliferation was also observed in 500 nmol/L TBBPA treatment group (Figure 4B). When combined with T3, the lowest concentration of TBBPA resulted in more EdU-labeled cells, some of which migrated far away from the SVZ in comparison with T3 treatment. In contrast, decreased EdU-labeled cells and ventricle size in telencephalons were observed in the co-treatment with 500 nmol/L TBBPA and T3 compared to T3 treatment, and even appeared to be comparable with those of controls, showing 500 nmol/L TBBPA antagonized and even counteracted T3 actions. The median concentration (50 nmol/L) appeared to have no pronounced effect on T3-induced cell proliferation. As a whole, TBBPA treatment affected T3-induced cell proliferation in *Xenopus* brains in a biphasic concentration-response manner. In the 500 nmol/L TBBPA treatment alone, there were more EdU-marked cells than the control, showing that 500 nmol/L TBBPA increased cell proliferation, despite being less relative to T3 treatment.

### 2.4. TBBPA Affects Neurodifferentiation in the Telencephalon in the Presence of T3

**Figure 5:**
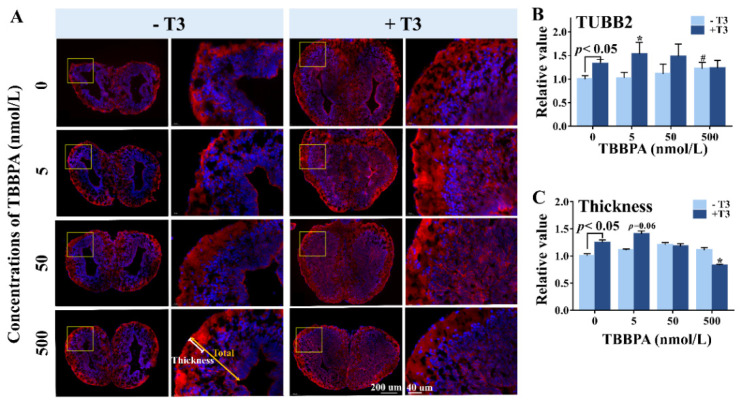
The effect of TBBPA on neurodifferentiation in the brain of NF stage 52 *Xenopus laevis* tadpoles exposed to a series of concentrations of TBBPA in the absence or presence of 1 nmol/L T3. (**A**) the immunofluorescence images of telencephalons retained TUBB2 (red) and DAPI (blue). (**B**) the relative fluorescence intensity of TUBB2, normalized by the control group. (**C**) The relative thickness of the outer neuropil. The data were normalized by the mean value of the solvent control. Data are shown as mean ± SD. # indicates significant differences between TBBPA treatment and solvent control treatment (*p* < 0.05). * indicates significant differences between TBBPA + T3 treatment and T3 treatment (*p* < 0.05). The experiment was repeated three times using tadpoles from different sets of adults. The results were consistent among the three independent experiments.

By immunofluorescence (IF) staining, we detected neuronal marker TUBB2 in the telencephalon to characterize neuronal differentiation in *Xenopus* brain (Figure 5A). Compared with controls, T3-treated brains exhibited significantly higher TUBB2 expression (Figure 5B), with thicker outer neuropil of the telencephalon (Figure 5C), indicating that T3 promoted neurodifferentiation in the brain. Similarly, 50 nmol/L and 500 nmol/L TBBPA-treated brains also exhibit stronger TUBB2 staining and thicker outer neuropil, despite the lack of a statistical difference between the 50 nmol/L TBBPA group and the control, suggesting a stimulatory effect of higher concentrations of TBBPA on neurodifferentiation. In the presence of T3, 5 nmol/L TBBPA led to stronger TUBB2 staining in comparison with T3, along with increased thicknesses of the outer neuropil (Figure 5B,C). However, 500 nmol/L TBBPA antagonized T3 action on neurodifferentiation, which was characterized by thinner outer neuropil and a decreasing trend of TUBB2 expression—combined with T3, while 50 nmol/L TBBPA had no significant effects on T3-induced TUBB2 expression and thickening of the outer neuropil. Overall, 5–500 nmol/L TBBPA affected T3-induced neurodifferentiation in *Xenopus* brains in a biphasic concentration-response manner.

### 2.5. TBBPA Disturbs Brain Development in Spontaneous Metamorphosis Assay

**Figure 6:**
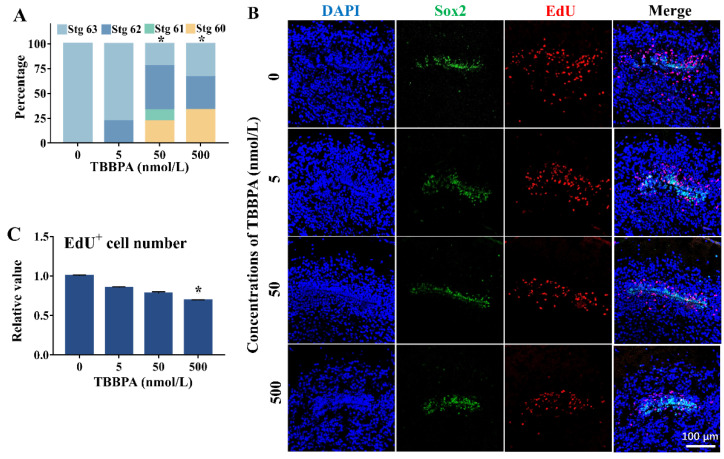
Effects of tetrabromobisphenol A (TBBPA) on spontaneous metamorphosis of *Xenopus laevis* at the metamorphic climax. (**A**) Percentages of tadpoles at different stages after a six-day exposure. * indicates a significant difference between TBBPA treatment and the solvent control group (*p* < 0.05). (**B**) Immunofluorescence images for telencephalons of NF 63 tadpoles. EdU labeled proliferating cells and migrating cells. Sox2 outlined the ventricle zone. (**C**) The ratio of EdU positive cells number. The data were normalized by the mean value of the solvent control tadpoles. * indicates a significant difference between TBBPA treatment and the solvent control group (*p* < 0.05).

Following the TIXMA, the effects of TBBPA on spontaneous metamorphosis and brain development were investigated. After six-day treatment since stage 58, all the control tadpoles reached stage 63, whereas some of the TBBPA-treated tadpoles remained at earlier developmental stages. Statistical analysis revealed significant inhibitory effects of 50–500 nmol/L TBBPA on spontaneous metamorphosis (Figure 6A). At stage 63, as SOX2 labeled the cells in the SVZ of the telencephalon, the EdU-labeled proliferating cells in the zone and its surrounding appeared to be concentration-dependently less in TBBPA-treated animals compared with the controls (Figure 6B), with a significant difference between 500 nmol/L TBBPA group and the control group (Figure 6C). Moreover, there were fewer EdU-labeled proliferating cells far away from the SVZ in the TBBPA-treated brains than controls. These observations indicate that TBBPA inhibited brain development at the metamorphic climax.

## 3. Discussion

In this study, we employed *X. laevis*, a TH-dependent developmental model, to address the influences of TBBPA on brain development by interfering with TH signaling. In the TIXMA, two days of TBBPA treatment alone did not alter TH-response gene (*thrb*, *thibz*, *klf9*, *mmp13*, *st3*, and *tgm2*) expression in brains of NF stage 52 *Xenopus*, implying the lack of TH signaling disruption at this stage, which differed from potential TH signaling agonism observed in *Xenopus* intestines and hindlimbs [13]. In fact, previous studies generally reported TBBPA as a TH signaling antagonist but not as a TH signaling agonist [24]. However, here, we found that 500 nmol/L TBBPA treatment for four days decreased the DL/BL values and increased cell proliferation and neurodifferentiation in the telencephalon, indicating a stimulatory effect on brain development at pre-metamorphic stages. Given the lack of TH signaling disruption [25,26], it is concluded that the stimulatory effect of TBBPA on brain development at pre-metamorphic stages is not associated with TH signaling. Maybe, there are other signaling pathways, such as Notch/Wnt signaling, that are involved in brain development [27], contributing to the stimulatory effect of TBBPA on tadpole brain development at pre-metamorphic stages. Indeed, TBBPA was reported to affect neural differentiation of embryonic stem cells and neural stem cells by partially disturbing Notch and Wnt signaling [28,29].

In this study, importantly, we found that in the presence of T3, 500 nmol/L TBBPA antagonized T3-induced TH-response gene expression, but 5 nmol/L TBBPA exerted stimulatory effects on T3 actions, showing an apparently biphasic concentration-response manner. These results agree with our previous observations in *Xenopus* intestines treated with TBBPA [13]. Recently, we found that bisphenol F and bisphenol A also affected T3-induced TH-response gene expression in *Xenopus* brains at pre-metamorphic stages in a biphasic concentration-response manner [21]. Given the strong evidence that TBBPA can act as a TH signaling antagonist in vitro [24], the antagonistic effect of 500 nmol/L TBBPA on T3-induced TH-response gene expression implies its TH signaling antagonism in *Xenopus* brains. As for the stimulatory effects of 5–50 nmol/L TBBPA on T3-induced TH-response gene expression, we speculate that some specific TH-dependent signaling pathways could have participated in the regulation of the TH gene expression response by TBBPA, which needs further studies. Overall, the effects of TBBPA on T3-induced TH-response gene expression in *Xenopus* brains at pre-metamorphic stages strongly indicate that TBBPA could interfere with TH-dependent signaling pathways.

Furthermore, in the TIXMA, TBBPA affected T3-induced brain morphological changes, cell proliferation, and neurodifferentiation. These findings reveal that TBBPA disrupted TH-dependent brain development. Notably, the effects of TBBPA on TH-dependent brain development were in a biphasic concentration-response manner, i.e., 5 nmol/L TBBPA exerted agonistic effects, whereas 500 nmol/L exerted antagonistic effects, which correspond to TH-response gene expression in the biphasic concentration-response manner. However, 50 nmol/L TBBPA antagonized T3-induced brain development, but it simulated T3-induced TH-response gene expression. Overall, the biphasic effects of TBBPA at the highest and lowest concentration on T3 actions in TH-response gene expression were in agreement with their effects on phenotype. However, we cannot give a reasonable explication for the inconsistent effects of the median concentration on TH-response gene expression and brain development, possibly suggesting complex mechanisms involving other signaling pathway(s) besides TH signaling.

T3-induced metamorphosis of *Xenopus* at pre-metamorphic stages can simulate TH-regulated development at the metamorphic climax, at which tadpoles contain high endogenous TH levels. Based on the findings that TBBPA affected T3-induced brain development, the effects of TBBPA on TH-dependent brain development at the metamorphic climax were further investigated. As expected, 50–500 nmol/L TBBPA significantly inhibited metamorphic development and brain development, which agrees with the antagonistic effects of higher concentrations on T3-induced brain development. At the metamorphic climax, however, the lowest concentration of TBBPA did not promote brain development but exhibited an inhibitory trend in terms of cell proliferation and migration in brains, which is inconsistent with the results in the TIXMA. Nevertheless, all findings strongly demonstrate that TBBPA has significant effects on TH-dependent and TH-independent brain development. Given the evolutionary conservation of TH signaling and its roles in brain development [30,31], we infer that TBBPA could exert similar effects on brain development in other vertebrates, including mammals. However, the extrapolation of our findings from *Xenopus* to mammal species warrants cautions due to the difference between waterborne exposure and general oral administration [32,33,34]. Nevertheless, our study supports previous data that TBBPA exerted significant effects on the nervous system in the literature [35].

## 4. Materials and Methods

### 4.1. Chemicals

3,3′,5-triiodo-Lthyronine (T3) from Geel Belgium (New Jersey, USA) was dissolved in ultrapure water (with NaOH) to prepare a 10 mmol/L stock solution. TBBPA from Tokyo Chemical Industry Co. Ltd. (Tokyo, Japan) was dissolved in DMSO to prepare 0.5 mol/L TBBPA stock solution. EdU (5-ethynyl-2′-deoxyuridine) from RiboBio Co. (Guangzhou, China) were dissolved in ultrapure water to prepare 2 mg/mL stock solution. The β2-tubulin (7B9) antibody (SC-47751) and anti-SOX2 antibody (ab97959) were from Santa Cruz Biotechnology (California, USA) and Abcam (Cambridge, UK), respectively. Cy3^TM^ goat anti-mouse IgG (H+L) (A10521) secondary antibody and Cy3^TM^ goat anti-rabbit IgG (H+L) (A10520) were from Invitrogen (Carlsbad, California USA). RNA extraction kit was from Bio Teke Corporation (Beijing, China). Quantscript RT Kit and RealMasterMix (SYBR Green) Kit were from Tiangen (Beijing, China). PCR primers were synthesized by the BGI group (Beijing, China).

### 4.2. Animal Housing

*Xenopus* frogs were initially obtained from Nasco (Fort Atkins, WI, USA). Frogs and tadpoles were raised as described previously [36]. Fertilized eggs were obtained by injecting HCG into a pair of adult frogs and incubating them in dechlorinated tap water at 21–22 °C. Tadpoles were raised in a flow-through system (Esen, Beijing, China) at NF stage 46, staged according to the Nieuwkoop and Faber system [37].

### 4.3. T3-Induced Xenopus Metamorphosis Assay (TIXMA)

At the beginning of exposure, NF stage 52 tadpoles received an intracerebral injection of 0.5 μL of EdU (2 mg/mL) to label proliferating cells. Then tadpoles were randomly transformed into 4 L glass tanks for 5, 50, 500 nmol/L TBBPA exposure alone or combined exposure with 1 nmol/L T3, with 0.001% DMSO as the control. Three replicate tanks with six tadpoles per tank were employed for each treatment. Experiment water was renewed daily.

Following two days of exposure, tadpoles were anesthetized in MS-222 (100 mg/L), and each brain was collected for RT-qPCR analysis. After four days, tadpoles were weighed and photographed for gross morphologic analysis. Then each brain was sampled to fix in MEMFA solution (0.1 mol/L MOPS, 2 mmol/L ethyleneglycoltetraacetic acid, 1 mmol/L MgSO_4_, 3.7% formaldehyde, pH 7.4) for immunofluorescence (IF) staining and EdU labeling. TIXMA was repeated three times using the offspring from three different sets of adults.

### 4.4. Spontaneous Metamorphosis Assay

To verify the simulation of T3-induced metamorphosis, the spontaneous metamorphosis assay was conducted. NF stage 58 tadpoles at the early metamorphic climax received one intracerebral injection of 1.5 μL EdU (2 mg/mL) before exposure. Then tadpoles were treated with TBBPA (5, 50, and 500 nmol/L) in 10 L glass tanks as described above. Three replicate tanks and 12 tadpoles were employed for each treatment group. Experiment water was renewed every other daily. After six days, the developmental stage of each tadpole was recorded and statistically analyzed by SPSS, and brains were collected for IF and EdU staining.

### 4.5. RNA Extraction and RT-qPCR Analysis

The expression of six typical TH-response genes (*thibz*, *thrb*, *klf9*, *st3*, *mmp13*, and *tgm2*) was measured by RT-PCR. The *rpl8* was used as a housekeeping gene [19,38], the expression of *rpl8* was not affected by all treatments. Total RNA extraction, RT-qPCR analysis, and primers information were described in the Supplementary Materials [39,40,41].

### 4.6. Analysis for Gross Morphology

The morphological parameters for tadpoles were measured by the Image J software (1.52 a), including hindlimb length (HLL), head area, unilateral brain width (ULBW), and brain length (BL) [19], diencephalon length (DL), and diencephalon thickness (DT). Each parameter was normalized by the mean value of the control.

### 4.7. IF Staining

The neuronal marker β2-tubulin (TUBB2) was chosen to characterize neurodifferentiation by IF staining, with the stem cell marker SOX2 for labeling the ventricle area. The detailed procedures were described in Supporting Information. Confocal images were obtained on the Leica TCS SP5 and analyzed with Leica LAS AF Lite (Leica Microsystems CMS GmbH). The assay ensured no signal in the negative control. Three telencephalon sections per brain were analyzed by Image J; the neuronal differentiation was quantified by the intensity ratio of the TUBB2 to DAPI. The intensity of each picture was measured by spitting the channel, adjusting the threshold, and measuring to record the Mean value in Image J. The specific value of each exposure group was normalized by the control.

The relative thickness of the outer neuropil was defined by the ratio of the neuropil thickness to the length from the SVZ to the brain edge along the neurite direction. The length was measured using Image J. Three images were analyzed for each brain, with at least three brains for every treatment. The relative value was normalized by the control group.

### 4.8. EdU Proliferation Assay

EdU was injected as described in TIXMA and spontaneous metamorphosis assay. The brains were fixed, dehydrated, and transversely sectioned at 10 μm, with the same procedure for IF staining. Three animals from three replicate tanks were analyzed for each treatment. For EdU labeling, the Cell-LightTM Apollo 567 Stain Kit (Guangzhou RiboBio Co. Ltd. Guangzhou, China) was used according to the manufacturer’s technical information. The assay ensured no signal in the negative control. Confocal images were obtained as described previously. The number of EdU positive cells and the total number of cells (marked by DAPI) were counted manually and divided to get the cell proliferation ratio. Three images were counted in every treatment group, and the relative value was normalized by the control group.

### 4.9. Data Analysis

RT-qPCR data were present as means ± standard error of the mean (SEM), other quantitative data were shown as means ± standard deviation (SD). SPSS software v 18.0 (USA) was used for statistical analysis with the tank as the statistical unit. Two-way analysis of variance (ANOVA) followed by Dunnett’s test was employed for all data analysis, except the Chi-square test for the development stage distribution of spontaneous metamorphosis assay among TBBPA treatments.

## 5. Conclusions

Our study demonstrates that TBBPA could interfere with TH-signaling and subsequently disrupt TH-dependent brain development in *Xenopus*. In addition, TBBPA could also disrupt brain development, possibly via other signaling pathways besides TH signaling at specific developmental stages. Our study enhances the understanding of the neurodevelopmental effects of TBBPA on brain development in vertebrates.

## Data Availability

Not applicable.

## Supplementary Materials

The methods of total RNA extraction and immunofluorescence staining were described in the Supplementary Materials. Details of primers sequences of all tested genes used for qPCR (Table S1). Table S1: Primers sequences of all tested genes used for qPCR and related information.
