# Supplementary material for: Tetrabromobisphenol A Disturbs Brain Development in Both Thyroid Hormone-Dependent and -Independent Manners in Xenopus laevis
Source: Molecules. 2021 Dec 31;27(1):249. doi: 10.3390/molecules27010249 (PMC8746619; doi:10.3390/molecules27010249)
Supplement: Supplementary file 1 [file molecules-27-00249-s001.zip › molecules-1498465-SI.pdf]

molecules MDPI

Supplementary Materials and Methods

Tetrabromobisphenol A disturbs brain development in both thyroid hormone-dependent and -independent manners in *Xenopus laevis*

Mengqi Dong[1,2], Yuanyuan Li[1,2], Min Zhu[1,2], Jinbo Li[1,2], Zhanfen Qin[1,2,*]

1 State Key Laboratory of Environmental Chemistry and Ecotoxicology, Research Center for Eco-Environmental Sciences, Chinese Academy of Sciences, Beijing, 100085, China

2 University of Chinese Academy of Sciences, Beijing, 100049, China

Zhanfen Qin (✉)

Address: State Key Laboratory of Environmental Chemistry and Ecotoxicology, Research Center for Eco-Environmental Sciences, Chinese Academy of Sciences, 18 Shuangqing Road, P.O. Box 2871, Beijing 100085, China

Telephone: 86-10-62919177. Fax: 86-10-62923563.

E-mail: qinzhanfen@rcees.ac.cn (Zhanfen Qin)

1. Total RNA extraction methods

Total RNA was extracted from *X. laevis* brains by Automatic Nucleic Acid Extraction Apparatus (Bio Teke, Beijing, China) and the first-strand cDNA was synthesized from 1000 ng total RNA using the Fast RT Kit according to the manufacturer's instructions. RNA quality was examined by electrophoresis (DYCP-31F, Six One Instrument Factory, Beijing) on G-red nucleic acid dye-stained 1% agarose gels and by A260 nm/A280 nm ratio (Nanodrop ND-1000, Nano-Drop, Wilmington, USA) ranging from 1.8 to 2.0. The cDNA was stored at - 20 °C until further gene expression analysis by qPCR.

cDNA was amplified in duplicates with a reaction volume of 10 µl, consisting of 5 µl of SuperReal PreMix Plus, 0.3 µl of each primer, 3.4 µl water and 1 µl of diluted cDNA template using a Light Cycler 480 system

(Roche, Switzerland) under the following conditions: 95 °C for 15 min, 40 cycles at 95°C for 10 s, annealing at different temperatures (shown in Table 1) for 20 s, and 72°C for 20 s. Ribosomal protein L8 (*rpl8*) was used as a housekeeping gene to normalize mRNA expression, which is a frequently used reference gene for normalizing gene expression in amphibians in previous studies (Gunderson et al., 2011; Hogan et al., 2007). And in this study, the expression of *rpl8* was not affected by the TBBPA or TBBPA+T3 treatment. All PCR primers for concerned genes are listed in Table S1, including six TH-response genes (kruppel-like factor 9, *klf9*; transglutaminase 2, *tgm2*; matrix metallopeptidase 13, *mmp13*; thyroid hormone induced bZip protein, *thibz*; stromelysin-3, *st3* and thyroid hormone receptor beta, *thrb*). The fold changes of relative gene expression data using qPCR were determined by the 2−ΔΔCt methods (Livak and Schmittgen, 2001).

Table S1. Primers sequences of all tested genes used for qPCR and related information.

Gene	Species	Primer sequences	Annealing temperature (°C)
rpl8.S	*Xenopus*	F: CCGTGGTGTGGCTATGAATC<br>R: TACGACGAGCAGCAATAAGAC	60
thibz.S	*Xenopus*	F: CCACCTCCACAGAATCAGCAG<br>R: AGAAGTGTTCCGACAGCCAAG	60
mmp13.S	*Xenopus*	F: CCTTGTCAGTGCTTGTCCTATC<br>R: TCCTGGTGTCAGTTCAGAGTC	60
thrb.S	*Xenopus*	F: GAGATGGCAGTGACAAGG<br>R: CAAGGCGACTTCGGTATC	58
tgm2.S	*Xenopus*	F: AACAGGTCCATGCCCGTCTG<br>R: GTGCCTCCATCCGTGCTCTT	62
st3.L	*Xenopus*	F: CCTCTGTCATACACTTACCTT<br>R: TGAACCGTGAGCATTGAG	62
klf9.L	*Xenopus*	F: GTGGCCACTTGATTTCCCCT<br>R: AAAGACACAAAACAGCGGCG	64

Rpl8, ribosomal protein L8; *thrb*, thyroid hormone receptor beta; *klf9*, kruppel-like factor 9; *tgm2*, transglutaminase 2; *mmp13*, matrix metallopeptidase 13; *thibz*, thyroid hormone induced bZip protein; *st3*, stromelysin-3.

2. Immunofluorescence staining

The fixed brains were gradually dehydrated by immersing in 15% and 30% sucrose solution in phosphate-buffered saline (PBS), and finally embedded in OCT. The brain was transversely sectioned for 10 μm on a freezing microtome (MICROM, Walldorf, Germany) for subsequent immunofluorescence staining and EdU

proliferation assay. Slides were rinsed with PBS, followed by permeabilization with 0.1% Triton-X100 solution for 15 min. Then, slides were rinsed in PBS and incubated with the anti-β2-Tubulin antibody (1:500) or anti-Sox2 antibody (1:500) at 4°C overnight. After 3 times rinsing by PBS, the slides were incubated with the cy3-conjugated secondary antibody (1:1000) for 90 min at room temperature, followed by incubated with DAPI for 1 min to visualize cell nuclei. After rinsed by PBS 3 times, the slides were sealed by a cover glass. Confocal images were taken on the Leica TCS SP5 confocal microscope and images were analyzed with Leica LAS AF Lite (Leica Microsystems CMS GmbH). The assay ensured no signal in the negative control. Three telencephalon sections of each brain, which in the same location, were analyzed using Image J (1.8.0), with the ratio of the β2-Tubulin intensity to the DAPI intensity to quantify neurodifferentiation. The specific value of each exposure group was normalized by the mean value for the control.

References

Gunderson, M P, Veldhoen, N, Skirrow, R C, Macnab, M K, Ding, W, van Aggelen, G, Helbing, C C, 2011. Effect of low dose exposure to the herbicide atrazine and its metabolite on cytochrome P450 aromatase and steroidogenic factor-1 mRNA levels in the brain of premetamorphic bullfrog tadpoles (Rana catesbeiana). Aquat Toxicol 102:31-38.

Hogan, N S, Crump, K L, Duarte, P, Lean, D R, Trudeau, V L, 2007. Hormone cross-regulation in the tadpole brain: developmental expression profiles and effect of T3 exposure on thyroid hormone- and estrogen-responsive genes in Rana pipiens. Gen Comp Endocrinol 154:5-15.

Livak, K J, Schmittgen, T D, 2001. Analysis of relative gene expression data using real-time quantitative PCR and the 2(-Delta Delta C(T)) Method. Methods 25:402-408.
